# How Do Metal Ion Levels Change over Time in Hip Resurfacing Patients? A Cohort Study

**DOI:** 10.1155/2014/291925

**Published:** 2014-12-14

**Authors:** Lucia Savarino, Matteo Cadossi, Eugenio Chiarello, Caterina Fotia, Michelina Greco, Nicola Baldini, Sandro Giannini

**Affiliations:** ^1^Laboratory for Orthopaedic Pathophysiology and Regenerative Medicine, Rizzoli Orthopaedic Institute, Via di Barbiano 1/10, 40136 Bologna, Italy; ^2^Department I of Orthopaedics and Traumatology, Rizzoli Orthopaedic Institute, Via Pupilli 1, 40136 Bologna, Italy; ^3^Bologna University, Via Foscolo 7, 40123 Bologna, Italy

## Abstract

Metal-on-metal hip resurfacing (MOM-HR) is offered as an alternative to traditional hip arthroplasty for young, active adults with advanced osteoarthritis. Nevertheless, concerns remain regarding wear and corrosion of the bearing surfaces and the resulting increase in metal ion levels. We evaluated three cohorts of patients with Birmingham hip resurfacing (BHR) at an average follow-up of 2, 5, and 9 years. We asked whether there would be differences in ion levels between the cohorts and inside the gender. Nineteen patients were prospectively analyzed. The correlation with clinical-radiographic data was also performed. Chromium, cobalt, nickel, and molybdenum concentrations were measured by atomic absorption spectrophotometry. Chromium and cobalt levels demonstrated a tendency to decrease over time. Such tendency was present only in females. An inverse correlation between chromium, implant size, and Harris hip score was present at short term; it disappeared over time together with the decreased ion levels. The prospective analysis showed that, although metal ion levels remained fairly constant within each patient, there was a relatively large variation between subjects, so mean data in this scenario must be interpreted with caution. The chronic high exposure should be carefully considered during implant selection, particularly in young subjects, and a stricter monitoring is mandatory.

## 1. Introduction

Total hip arthroplasty (THA) leads to a marked improvement in the quality of life of patients who suffer from severe forms of hip diseases. Modern prosthetic design must address both the low-demand requirements of the elderly patient and the work and leisure aspirations of the younger patient. Metal-on-metal (MOM) articulations were introduced in order to overcome the drawbacks of polyethylene wear debris, thus reducing the incidence of periprosthetic osteolysis. Moreover, large diameter femoral heads are possible with MOM articulations leading to wider range of motion, increased stability, and lower dislocation rate. Hip resurfacing (HR) offers additional advantages such as bone stock preservation, lack of stress shielding [1], better functional outcomes [2–4], and resumption of sporting activity [2, 5]. HR represents a viable treatment option especially in young male with a femoral component that is >50 mm in size, with a reported revision rate at 10 years which compares favorably with conventional THA [6]. Nevertheless, concerns remain regarding metal ion level increase [7]. There have been two recent published reports of four patients in total who had high serum cobalt and/or chromium levels following MOM-THA and then developed symptoms that could indicate systemic metal toxicity [8, 9]. These findings potentially add a new dimension to the management of patients with MOM bearings regarding the indications for and timing of revision surgery. Potential effects of a chronic exposure to raised ion levels, such as chromosomal damage, teratological effects, and malignancy [10, 11], have not been yet clarified. In previous studies, concerning different implant designs and follow-ups, contradictory findings have been reported [12–16].

Homogeneous groups need to be analyzed over extended periods in order to identify any trends of the metal release over time and to establish safety levels especially in patients with a long life expectancy. So, our purpose was to evaluate how serum metal ion levels behave over time in three cohorts of subjects with unilateral Birmingham hip resurfacing at early-, medium-, and long-term follow-up. The secondary outcome was to verify if a correlation existed between ion release and other parameters, such as implant size and acetabular component inclination angle, at the three follow-ups.

## 2. Methods

### 2.1. Patients and Implant Design

A retrospective cohort study about the systemic distribution of metal ions in patients with well-fixed Birmingham hip resurfacing (Smith and Nephew, Memphis, TN, USA) was performed. The Institutional Ethics Committee on Human Research approved the study (prot. 0032742/IOR-05/11/2011) and the subjects signed informed consent forms to participate.

Fifty-five cases were identified from 2002 to 2012 and split into three follow-up cohorts: group 1 (2004-2005, mean 2-year follow-up) was constituted by fourteen subjects, group 2 (2008-2009, mean 5-year follow-up) by nineteen cases [15], and group 3 (2010-2011, mean 9-year follow-up) by twenty-two cases (Table 1). We excluded subjects with infection, malignancy, radiographic signs of loosening and/or osteolysis, and environmental or occupational chemical exposure. Also the ingestion of drugs containing metal ions was considered as exclusion criteria, as was renal impairment. Furthermore, nineteen cases out of fifty-five were prospectively analyzed. One patient had a bilateral implant since the beginning and other two patients required resurfacing of the contralateral hip during the study period. These patients were excluded from the statistical analysis; nevertheless, they are addressed separately in the discussion. Functional outcome was assessed at the follow-up visits by using the 10-UCLA scale, on the basis of information about lifestyles and activity level; Harris hip score (HHS) and plain radiographs of the operated hip were also taken. Forty-eight healthy blood donors (37 males and 11 females, 42 yrs ± 2 yrs age, range 20–71 yrs) who were not receiving medication and who did not have metal implants were used for reference values.

Birmingham hip resurfacing (BHR) was manufactured from a Co-Cr alloy: chromium (Cr) 26.5–30%, nickel (Ni) 0-1%, molybdenum (Mo) 4.5–7%, manganese (Mn) 0-1%, carbon (C) 0–0.35%, silicon (Si) 0-1%, iron (Fe) 0-1%, and cobalt (Co) balance, with a high level of carbon as a cast with no heat treatment [17]. It had a coverage angle ranging from 158 to 166° and a diametral clearance of approximately 210 *μ*m. The acetabular component was uncemented, whereas the femoral component was cemented.

All implants remained* in situ* throughout the course of the study. Implant size (acetabular and head diameters), acetabular component inclination angle [18], and body mass index (BMI) were recorded, too.

### 2.2. Metal Ion Analysis

Chromium, cobalt, nickel, and molybdenum ions were measured in the serum. Whole peripheral blood was collected into metal-free Vacutainers (Becton Dickinson and Co, Meylan, France) from the antecubital veins of fasting subjects in the morning. To avoid contamination from the needle, the first 5 mL of blood withdrawn was discarded. The samples were centrifuged at 800 ×g, 10 min, 4°C, and frozen at –70°C until analysis. Serum chromium (Cr), cobalt (Co), nickel (Ni), and molybdenum (Mo) ions were measured by employing a graphite furnace atomic absorption spectrometer, equipped with double background correction Deuterium/Zeeman (Thermo Fisher ICE4000; Thermo Fisher, Cambridge, UK). Environmental and sampling contamination was avoided when determining ion content by using a dedicated room with efficient fume extraction and temperature monitoring. A clean bench area was reserved for solution preparation. Every item used from the time of sampling until analysis was regarded as a potential source of contamination and was used only after soaking in 2% HNO_3_ in twice-distilled and deionized water, followed by thorough rinsing in twice-distilled and deionized water. Each item was then checked using a nitric acid leaching test to ensure that it did not contain detectable amounts of the relevant trace elements. All the results were expressed as ng/mL (equivalent to micrograms per liter and parts per billion). Calibration was performed by applying the standard addition method and by using certified standard solutions at three concentrations for each element (National Institute of Standards and Technology, NIST, USA). The specimens were diluted with 0.1 vol. % HNO_3_, 0.05 vol. % Triton ×100 (Santa Cruz Biotechnology, Inc., Heidelberg, Germany), and magnesium nitrate as a matrix modifier and were analyzed as 15 *μ*L aliquots in triplicate. The accuracy and precision of the method were validated using SRM 1598 NIST human serum for all the elements. Additionally, UTAK (UTAK Laboratories Inc., Valencia, CA, USA) normal and high-range trace elements were used for Cr. Test repeatability was ensured by rejecting ion levels with a relative standard deviation greater than 10%. The detection limits for sample matrix were 0.06 ng/mL for Cr, 0.08 ng/mL for Co, 0.1 ng/mL for Ni, and 0.83 ng/mL for Mo. All the subjects having ion levels below the detection levels were adjusted to the detection limit values.

### 2.3. Statistics

Patient groups were matched for age, implant size, acetabular component inclination angle, BMI, HHS, and 10-UCLA scale, as verified by applying the Kruskal-Wallis test, as well as for primary pathology and gender, as evaluated by the Fisher exact test. Ion concentrations were expressed as arithmetic means plus and minus standard errors of the mean (m ± SE), minimum-maximum range, and median value. Since metal ion data are not normally distributed, nonparametric tests were used for analysis. Differences between the groups regarding ion release were evaluated using the Kruskal-Wallis test and the Mann-Whitney* U *test, depending on the number of variables considered. Furthermore, the groups were stratified by gender and the subgroups of males and females were compared with each other by using the Mann-Whitney* U* test, calculated according to the exact test for small samples; corrections were made for multiple comparisons.

The correlation between ion values and other parameters, that is, UCLA scale and HHS, implant size, acetabular component inclination angle, and BMI, was also calculated in the resurfacing groups, by using Spearman's *r* coefficient.

The Stat View statistical software system (version 5.01.0, SAS Institute Inc., Cary, NC, USA) was used to analyze the results and the level of significance was set at *P* < 0.05. The MedCalc v11.5.1 software (Broekstraat 52, 9030 Mariakerke, Belgium) was used to perform the Mann-Whitney* U* test, calculated according to the exact test.

## 3. Results

Patient cohorts did not show any significant difference regarding age (*P* = 0.27), gender (*P* = 0.14), and primary pathology (*P* = 0.56), which were not considered potentially confounding variables. Also HHS and the 10-UCLA scale evaluated at the follow-up visits did not show any significant difference between groups (Kruskal-Wallis test; *P* = 0.58 and *P* = 0.49, resp.), as well as acetabular and head diameters, acetabular component inclination angle, and BMI (Kruskal-Wallis test; *P* = 0.22, *P* = 0.73, *P* = 0.83, and *P* = 0.15, resp.).

Table 2 reports the ion values in the three groups of BHR patients, as whole cohorts and split by gender. The references ranges in healthy subjects for each ion are reported, too.

The Mann-Whitney* U* test, applied to highlight specific differences between groups, showed that the concentrations of Cr and Co were significantly higher (*P* = 0.001) than those of healthy subjects (Cr range: 0.06–0.67 ng/mL; Co range: 0.08–0.53 ng/mL) at each follow-up, whereas Ni values were higher than controls only at medium term (Ni range: 0.10–1.32 ng/mL) and returned within the normal range at long term. Mo concentrations were significantly different between groups, even if in most cases they were below the detection limit. These levels were always within the reference normal ranges reported by the Istituto Superiore di Sanità (Mo range: 0.20–2.75 ng/mL) [19] and were not further analyzed.

Comparing Co and Cr serum levels between the three cohorts of BHR patients with different follow-up no statistical differences were found. Nevertheless a tendency to decrease was observed over time.

The gender-based analysis reported in Table 2 showed that the tendency to decrease over time of Cr and Co levels was recorded only in females, who had, however, values above those of males at the three follow-ups. On the other side, males demonstrated an increase over time of Co concentration. Also Ni values rose significantly at medium term; then they significantly declined at long term. The peak of Ni at medium term was present regardless of gender.

A correlation between Cr and Co levels was found in all cohorts (*r* = 0.64 and *P* = 0.02; *r* = 0.76 and *P* = 0.001; *r* = 0.67 and *P* = 0.002, at short, medium, and long term, resp.), as well as between Cr and Ni at medium term (*r* = 0.49, *P* = 0037), by applying Spearman's *r* coefficient.

Acetabular component inclination angle, BMI, and 10-UCLA scale did not correlate with any ion concentrations. Conversely, an inverse significant correlation was shown between Cr levels and HHS, head, and acetabular implant size in the short-term group (*r* = −0.71 and *P* = 0.01; *r* = −0.61 and *P* = 0.03; *r* = −0.54 and *P* = 0.03, resp.). These correlations disappeared over time.

Nineteen subjects were analyzed prospectively (Table 3). Few patients showed a common trend with increased levels of Co and Cr from two to five years; then this steady state was maintained over time. Nevertheless different behaviors were observed among different patients. In some subjects levels declined, but in some others they gradually rose again up to ten years. One patient had a bilateral BHR since the beginning (case #17) and maintained similar ion levels from two- to five-year follow-up. Two other patients underwent contralateral surgery before the second follow-up (case #18 and case #19). Case #18, who was followed prospectively, showed a specific trend in ion concentrations: Co and Cr values nearly doubled when the second BHR was implanted, 6 months before the medium-term analysis, and then dropped at long term. Considering that the consistent rise during the first 18 months to two years after surgery has been widely attributed to the “running-in” period, when small irregularities on the bearing surfaces are worn down [16], a similar behavior may be expected for case #19 but unfortunately long-term results are missing.

## 4. Discussion

MOM-HR has gained popularity as an alternative to conventional THA in younger, active patients. However, high serum ion levels are a matter of concern. We asked whether there would be differences over time in serum ion concentrations between three cohorts of subjects with unilateral HR. Moreover, we aimed to verify if a correlation existed between ion release and other parameters, such as implant size and acetabular component inclination angle, at three follow-ups.

Many studies have reported raised systemic ion concentrations after HR, but usually ion levels have been monitored exclusively up to 24 months postoperatively and different implant design and alloy composition are described [20–22]. A few studies analyzed metal distribution in BHR and at longer follow-up: Daniel et al. [12, 13] undertook a longitudinal study over a period of six years and observed a significant Co and Cr increase at one year, followed by a decrease until the sixth year. DeSouza et al. [16] reported serum levels in a little number of patients with HR over a ten-year period; they found that metal levels increased during the first 18 months after surgery and then remained elevated and declined slowly for up to five years, but in some patients they appeared to start rising again five up to ten years. van der Straeten et al. [23] investigated ion levels in a series of BHR and found that Cr and Co levels decreased significantly from the initial assessment at a median of six years to the last assessment at a median of 11 years.

In our study three cohorts of patients with 2-, 5-, and 9-year follow-up, respectively, were analyzed. Differently from van der Straeten et al. [23] no significant differences in Cr and Co levels between these three cohorts were found, even if a tendency to decrease over time was observed. Moreover, we found an opposite behavior of metal levels between males and females. While females showed a decreasing trend for both Co and Cr, male population was characterized by increasing levels of Co through the study period. Although we do not have an explanation for this particular finding, we could hypothesize that the decreasing trend reflects the positive outcome of BHR wear, which may reach a steady state; on the contrary, a progressive increase of metal ion levels should be suspected as an early sign of implant loosening. The higher female values could depend on the smaller implant size, as it has been demonstrated that larger implants have a better performance, probably due to a larger diameter of the femoral head, which provides a better lubrication regimen [24]. Also, our finding of an inverse correlation between the highest Cr levels and the implant size strengthens this concept.

We showed also an inverse significant correlation between Cr levels and HHS at short term. A possible explanation for this result may be that a well-functioning hip resurfacing often reflects a well-positioned implant avoiding impingement phenomenon, which may lead to higher ion release. These correlations disappeared at the longer follow-ups, probably due to the tendency to decrease of ion levels. No correlation was found between ion concentrations and acetabular inclination. It is well known that acetabular component orientation in terms of both inclination and version affects metal ion levels [25]. Our findings might be explained taking into consideration that only 3 patients had an acetabular component inclination angle >55° which is likely to give rise to higher ion levels [1]. Case #11, who had the steeply inclined cup, had the highest Co and Cr concentrations. Dysplastic patients may have an excessive anteversion of the acetabular component due to the native acetabular deficiency. Even if there is a subtle difference in the three groups with a greater proportion having hip dysplasia in group 1 (35%) compared to 15 and 14% in the other two groups, most of the patients suffered from grade 1 dysplasia according to Crowe classification, with only one patient with a grade II dysplasia. By considering this, we believe that all acetabular components were well oriented with an anteversion ranging from 10 to 20°, as it was measured on cross table X-rays, when available. Nevertheless, this aspect must be considered as a potential confounding factor [26].

Similar other results [1] no correlation was found between metal ion levels and activity level.

With regard to the other ions, Mo values were always within the reference normal ranges. Mo is present in low concentration in the Co-based alloy and is rapidly transported to the urine and eliminated from the body [27]. Consequently, it was not considered a major concern in the biological response to MOM implants. Similarly, the significant Ni increase observed at medium term was not taken into consideration because only three cases exceeded the normal range.

The evaluation of the patients prospectively analyzed showed that, although ion levels remained fairly constant within any given patient, there was a relatively large variation between subjects. Even if it is well established to discuss the mean level in a specific cohort of patients, we agree with DeSouza et al. [16] that caution should be taken when interpreting data because around the mean there is considerable scatter.

Although it is clear that metal ions in resurfacing patients are higher than in healthy population, the clinical significance of these raised levels remains a matter of concern which led to diminished use and acceptance of MOM bearings. de Smet et al. [28] reported that serum Cr levels of >17 *μ*g/L (ng/mL) and Co levels of >19 *μ*g/L (ng/mL) are strongly associated with metallosis and should be considered as an index of implant loosening or malfunctioning. On the contrary, there is limited information about the range of acceptable ion concentrations and where toxicity is introduced. The relevance of chronic low-grade exposure to these ions and the limit values for health to be impaired by such ions by an inner route are still unknown. Some authors reported that serum ion level for Cr and Co less than 10 *μ*g/L (ng/mL) does not exclude the risk of systemic toxicity, or carcinogenicity [29], even if other epidemiological findings showed that either the incidence of cancer was reduced in patients with MoM resurfacing or no difference had been found [30–32]. In our opinion, considering the long latency period of cancers, the risk of chronic exposure to metal ions, although at lower levels, should not be disregarded, particularly in the female young population. In conclusion, even if patients with BHR implants demonstrated an average tendency to ion decrease over time, the large variation of the serum concentrations between subjects requires a stricter monitoring, in order to determine the true incidence of local and systemic complications and to undertake prevention measures.

## Figures and Tables

**Table 1:** Profile of the cohorts of subject with metal-on-metal hip resurfacing at short (group  1), medium (group  2), and long term (group  3). Values are expressed as mean ± standard error (median) and minimum–maximum range.

Variable	Group 1	Group 2	Group 3
Gender			
Males	8	10	15
Females	6	9	7

Age (years)	49 ± 3 (44) 26–75	53 ± 2 (55) 30–65	53 ± 2 (53) 26–73

Diagnosis			
Osteoarthritis	7	14	16
Congenital hip dysplasia	5	3	3
Rheumatoid arthritis	1	—	1
Trauma	1		—
Necrosis	—	2	—
Epiphysiolysis	—	—	1
Coxitis	—	—	1

Follow-up (years)	2 ± 0.1 (2.0) 1.3–2.7	5 ± 0.05 (4.7) 4.6–5.2	9 ± 0.2 (8.5) 8.0–11.0

Body mass index	29 ± 2 (28) 23–40	27 ± 1 (27) 22–34	25 ± 1 (25) 17–32

Activity level (10-UCLA scale)	7.8 ± 0.5 (8.4) 5.9–10	6.9 ± 0.4 (6.0) 5.0–10.0	7.3 ± 0.4 (7.0) 3.0–10.0

Head diameter	48.7 ± 1.6 (46) 38–60	46.8 ± 3.2 (46) 38–54	48.9 ± 0.96 (50) 38–54
Acetabular component diameter	54.9 ± 1.2 (54) 46–60	54.4 ± 3.1 (58) 46–60	56.2 ± 0.93 (56) 46–62

Cup inclination angle	45 ± 2.2 (45) 30–59	46.0 ± 1.9 (45) 30–60	45 ± 1.2 (45) 32–58

Harris hip score	95 ± 1.8 (100) 83–100	95 ± 1.1 (96) 88–100	94.8 ± 1.6 (98) 69.7–100

**Table 2:** Ion values expressed as ng/mL (nanograms per milliliter, mean ± standard error, median, and minimum–maximum range) in metal-on-metal hip resurfacing at short [15], medium [15] and long term, as whole cohorts and split by gender. Reference ranges are reported, too.

	Group** **1 [15] (2-year follow-up)	Group** **2 (5-year follow-up)	Group** **3 (9-year follow-up)	*P*	^*^ *P*	^**^ *P*
Chromium	Reference range: 0.06–0.67 ng/mL					
Whole cohort	2.18 ± 0.51 (1.53) 0.69–7.24	2.26 ± 0.49 (1.63) 0.49–10.47	1.82 ± 0.33 (1.27) 0.56–6.19	0.64	0.38	0.17
Males	1.12 ± 0.12 (1.09) 0.69–1.79	1.44 ± 0.17 (1.30) 0.88–2.47	1.49 ± 0.36 (1.06) 0.56–6.19	0.16	0.79	0.46
Females	3.61 ± 0.93 (2.46) 1.73–7.24	3.17 ± 0.96 (2.36) 0.49–10.47	2.52 ± 0.69 (2.06) 0.93–6.01	0.63	0.20	0.56

Cobalt	Reference range: 0.08–0.59 ng/mL					
Whole cohort	1.17 ± 0.61 (0.55) 0.08–8.96	1.13 ± 0.27 (0.72) 0.30–5.60	0.90 ± 0.12 (0.77) 0.29–2.45	0.14	0.12	0.86
Males	0.39 ± 0.09 (0.39) 0.08–0.76	0.82 ± 0.18 (0.63) 0.30–2.27	0.72 ± 0.08 (0.68) 0.29–1.54	**0.05**	**0.01**	0.75
Females	2.22 ± 1.36 (0.85) 0.41–8.96	1.47 ± 0.53 (1.20) 0.30–5.60	1.28 ± 0.30 (0.93) 0.32–2.45	>0.1	0.89	0.67

Nickel	Reference range: 0.10–1.32 ng/mL					
Whole cohort	0.64 ± 0.06 (0.69) 0.10–0.99	0.95 ± 0.09 (0.93) 0.34–1.95	0.38 ± 0.06 (0.31) 0.10–1.50	0.01	0.001	<0.001
Males	0.62 ± 0.1 (0.72) 0.1–0.99	0.78 ± 0.09 (0.78) 0.34–1.25	0.40 ± 0.09 (0.32) 0.17–1.50	0.33	**0.04**	**0.001**
Females	0.67 ± 0.06 (0.66) 0.48–0.94	1.14 ± 0.15 (0.94) 0.72–1.95	0.35 ± 0.09 (0.29) 0.10–0.79	0.08	**0.03**	**0.002**

Molybdenum	Reference range: 0.20–2.75 ng/mL					
Whole population	0.89 ± 0.03 (0.83) 0.83–1.23	0.84 ± 0.01 (0.83) 0.83–0.96	0.85 ± 0.01 (0.83) 0.82–0.97	0.06	0.001	0.001

*P* = group 1 versus group 2, ^*^ *P* = group 1 versus group 3, and ^**^ *P* = group 2 versus group 3.

**Table 3:** Prospective clinical, radiographic, and biochemical evaluation of nineteen HR patients.

Code at ***≠*** follow-up	Gender	Age at surg.	Status^*^	2-year follow-up				5-year follow-up				9-year follow-up				Acetab. Ø mm	Head Ø mm	Cup inclin. angle°	HHS	UCLA scale
				Cr	Co	Ni	Mo	Cr	Co	Ni	Mo	Cr	Co	Ni	Mo					
(1) 3712/4567	M	52	U	■	■	■	■	1.62	0.51	0.67	0.83	1.17	0.78	0.17	0.93	56	50	58^#^	92/97	5/6
(2) 2819/3546/4556	F	42	U	1.73	0.41	0.69	0.98	2.61	1.44	0.77	0.83	3.81	2.45	0.49	0.83	46	38	40	90/94/95	9/9/10
(3) 3561/4564	M	45	U	■	■	■	■	2.47	2.27	1.03	0.83	1.41	0.87	0.39	0.83	62	54	45	99	9/10
(4) 2808/4542	M	42	U	1.09	0.08	0.69	0.83	■	■	■	■	1.30	0.53	0.2	0.97	56	50	32	100	7
(5) 3559/4546	M	50	U	■	■	■	■	1.34	0.37	0.97	0.83	0.84	0.36	0.41	0.83	62	54	48	100/98	9/7
(6) 2817/3567	M	60	U	0.82	0.53	0.79	1.04	0.88	0.55	0.73	0.83	■	■	■	■	60	54	30	100	10
(7) 2812/3464/4547	M	55	U	0.88	0.23	0.55	0.83	1.16	0.52	0.51	0.96	0.86	0.77	0.3	0.83	60	54	46	100	9/8/7
(8) 3713/4585	M	61	U	■	■	■	■	1.00	0.83	0.51	0.83	1.01	0.999	0.32	0.83	62	54	47	96/100	6/10
(9) 2894/4554	M	42	U	1.08	0.11	0.1	0.83	■	■	■	■	1.06	0.68	0.24	0.83	52	46	46	100/98	8
(10) 2830/4583	F	37	U	2.08	0.58	0.94	0.83	■	■	■	■	1.24	0.64	0.79	0.83	54	46	43	90/97	9
(11) 2882/4559	F	73	U	7.24	8.96	0.48	1.23	■	■	■	■	28.03	32.24	0.47	1.59	52	46	59^#^	83/57	7
(12) 3664/4568	M	62	U	■	■	■	■	0.91	0.3	0.34	0.83	0.73	0.58	0.28	0.83	58	50	56	95/85	5/6
(13) 2845/4562	F	42	U	5.67	1.67	0.69	0.83	■	■	■	■	2.30	1.6	0.1	0.83	54	46	55	84/79	7
(14) 2844/3460/4544	F	57	U	2.36	0.74	0.6	0.83	1.78	1.36	0.94	0.83	0.93	0.93	0.23	0.91	48	42	42	86/90/70	6/6/3
(15) 2895/3714/4566	M	24	U	1.33	0.45	0.99	0.83	1.60	0.72	0.99	0.83	1.71	0.82	0.42	0.83	58	46	40	100/89/95	7/6/4
(16) 2713/4541	M	63	U	0.69	0.76	0.34	0.83	■	■	■	■	4.10	2.39	0.39	0.93	54	60	45	100/98	7
(17) **2816**/**3568** ^†^	F	59	B	**2.99^#^**	**1.62^#^**	**0.69^#^**	**1.3^#^**	**2.62^#^**	**2.06^#^**	**0.64^#^**	**∗∗^#^**	■	■	■	■	52	50	45	90/100	7
(18) 2839/**3544**/**4560** ^††^	F	47	U/B	2.57	0.96	0.63	0.83	**4.78^#^**	**2.89^#^**	**0.6^#^**	**∗∗^#^**	**2.98^#^**	**1.53^#^**	**0.61^#^**	**0.83^#^**	54	46	45	100/97/69	7/7/3
(19) 2810/**3558** ^†††^	M	39	U/B	1.79	0.63	0.74	0.83	**4.21^#^**	**2.38^#^**	**0.43^#^**	**∗∗^#^**	■	■	■	■	60	54	50	100/97	8/9

^†^Bilateral implant from 2 yrs and 5 yrs, ^††^one and four years after contralateral surgery, ^†††^one year after contralateral surgery, ^*^U: unilateral/B: bilateral, ^**^insufficient serum for Mo.
